# Assigning Transcriptomic Subtypes to Chronic Lymphocytic Leukemia Samples Using Nanopore RNA-Sequencing and Self-Organizing Maps

**DOI:** 10.3390/cancers17060964

**Published:** 2025-03-13

**Authors:** Arsen Arakelyan, Tamara Sirunyan, Gisane Khachatryan, Siras Hakobyan, Arpine Minasyan, Maria Nikoghosyan, Meline Hakobyan, Andranik Chavushyan, Gevorg Martirosyan, Yervand Hakobyan, Hans Binder

**Affiliations:** 1 Institute of Molecular Biology NAS RA, Yerevan 0014, Armeniasiras.hakobyan@abi.am (S.H.); maria.nikoghosyan@abi.am (M.N.);; 2 Institute of Biomedicine and Pharmacy, Russian-Armenian University, Yerevan 0051, Armenia; 3 Armenian Bioinformatics Institute, Yerevan 0014, Armenia; binder@izbi.uni-leipzig.de; 4 Hematology Center After Prof. R. Yeolyan MoH RA, Yerevan 0014, Armenia; 5 Interdisciplinary Center for Bioinformatics, Leipzig University, 04109 Leipzig, Germany

**Keywords:** chronic lymphocytic leukemia, transcriptome, nanopore sequencing, self-organizing maps, machine learning, transfer learning

## Abstract

**Background/Objectives:** Massively parallel sequencing technologies have advanced chronic lymphocytic leukemia (CLL) diagnostics and precision oncology. Illumina platforms, while offering robust performance, require substantial infrastructure investment and a large number of samples for cost-efficiency. Conversely, third-generation long-read nanopore sequencing from Oxford Nanopore Technologies (ONT) can significantly reduce sequencing costs, making it a valuable tool in resource-limited settings. However, nanopore sequencing faces challenges with lower accuracy and throughput than Illumina platforms, necessitating additional computational strategies. In this paper, we demonstrate that integrating publicly available short-read data with in-house generated ONT data, along with the application of machine learning approaches, enables the characterization of the CLL transcriptome landscape, the identification of clinically relevant molecular subtypes, and the assignment of these subtypes to nanopore-sequenced samples. **Methods:** Public Illumina RNA sequencing data for 608 CLL samples were obtained from the CLL-Map Portal. CLL transcriptome analysis, gene module identification, and transcriptomic subtype classification were performed using the oposSOM R package for high-dimensional data visualization with self-organizing maps. Eight CLL patients were recruited from the Hematology Center After Prof. R. Yeolyan (Yerevan, Armenia). Sequencing libraries were prepared from blood total RNA using the PCR-cDNA sequencing-barcoding kit (SQK-PCB109) following the manufacturer’s protocol and sequenced on an R9.4.1 flow cell for 24–48 h. Raw reads were converted to TPM values. These data were projected into the SOMs space using the supervised SOMs portrayal (supSOM) approach to predict the SOMs portrait of new samples using support vector machine regression. **Results:** The CLL transcriptomic landscape reveals disruptions in gene modules (spots) associated with T cell cytotoxicity, B and T cell activation, inflammation, cell cycle, DNA repair, proliferation, and splicing. A specific gene module contained genes associated with poor prognosis in CLL. Accordingly, CLL samples were classified into T-cell cytotoxic, immune, proliferative, splicing, and three mixed types: proliferative–immune, proliferative–splicing, and proliferative–immune–splicing. These transcriptomic subtypes were associated with survival orthogonal to gender and mutation status. Using supervised machine learning approaches, transcriptomic subtypes were assigned to patient samples sequenced with nanopore sequencing. **Conclusions:** This study demonstrates that the CLL transcriptome landscape can be parsed into functional modules, revealing distinct molecular subtypes based on proliferative and immune activity, with important implications for prognosis and treatment that are orthogonal to other molecular classifications. Additionally, the integration of nanopore sequencing with public datasets and machine learning offers a cost-effective approach to molecular subtyping and prognostic prediction, facilitating more accessible and personalized CLL care.

## Simple Summary

Chronic lymphocytic leukemia (CLL) is a type of blood cancer where accurate subtyping can enhance diagnosis and treatment. In this study, we integrated nanopore sequencing data with publicly available Illumina datasets and applied machine learning to identify distinct molecular subtypes of CLL. These subtypes were linked to patient survival, independent of genetic mutations or gender. Our findings suggest that combining nanopore sequencing with machine learning provides a cost-effective approach to classifying CLL cases and improving personalized treatment strategies supporting more accessible and personalized CLL care in resource-limited settings.

## 1. Introduction

Chronic lymphocytic leukemia (CLL) is one of the most prevalent cancers of bone marrow and blood [1]. The clinical course of CLL ranges from indolent to highly aggressive, with considerably different survival rates and prognoses [2]. While this clinical heterogeneity was attributed to somatic mutations in the heavy-chain variable region of immunoglobulin genes, more studies have now focused on transcriptomic or multi-omic stratification of CLL [3,4]. Early research on transcriptome analysis of CLL showed the presence of two transcriptional subtypes associated with clinical phenotypes of the diseases independent of IGHV mutation status [5]. Another transcriptomic study identified co-expressed gene networks related to disease relapse and survival [6]. A recent multi-omic molecular classification of CLL presented refined genomic subtypes, and new gene expression subtypes with independent prognostic values were discovered [7].

These advancements, among many others, have been made possible through the use of short-read next-generation sequencing (NGS). Furthermore, NGS is now widely integrated into the diagnostics and clinical management of CLL [8]. While its accuracy and reliability are well established, they come at the expense of substantial investments in capital, maintenance, and operational costs. Additionally, most current NGS platforms are cost-effective only when there is a large number of samples per run and a constant flow of samples. These limitations hinder the widespread adoption of NGS in low- and middle-income countries [9]. As a result, more affordable approaches like nanopore sequencing, developed by Oxford Nanopore Technologies (ONT), are gaining popularity as an alternative, helping to democratize access to sequencing technology for both research and clinical care [10]. Nanopore sequencing, while error-prone and lower in throughput than Illumina platforms, is suitable for gene expression-based classifications of hematological cancers [11]. However, current data volumes generated by nanopore sequencing alone may not be sufficient to build robust classifiers. Nonetheless, combining nanopore data with publicly available NGS datasets offers additional opportunities for its use in research and clinical applications. In this paper, we demonstrate that integrating publicly available short-read data with in-house generated ONT data, along with the application of machine learning approaches, enables the characterization of the CLL transcriptome landscape, the identification of clinically relevant molecular subtypes, and the assignment of these subtypes to nanopore-sequenced samples.

## 2. Materials and Methods

### 2.1. CLL Public Data Source

CLL RNA-sequencing data (CLLmap dataset) were downloaded from the CLL-Map Portal [7]. This dataset contains various-omic data for 1158 patients with CLL collected in 6 cohorts. RNA-sequencing data, represented as batch-adjusted transcript per million (TPM) values, were available for 608 patients.

### 2.2. Transcriptome Portrayal Using Self-Organizing Maps (SOMs)

Transcriptome portrayal with the CLLmap dataset was performed using the oposSOM R package [12]. Since the CLLmap dataset was composed of several patient cohorts [7], we performed batch correction of TPM values using the ComBat empirical Bayes approach [13], treating each cohort as a batch (Supplementary Figure S1). Then, TPM values were log-transformed (log2(TPM + 1)), sample-wise quantile normalized, and normalized gene-wise by subtracting the log expression value for a gene from the mean value of that gene across all samples. The resulting dataset was used for self-organizing maps (SOMs), which transformed the expression profiles of 32,378 genes in 608 samples into 2500 (50 × 50) gene clusters (metagenes) using the Euclidean distance metric by creating a data matrix of reduced dimensionality of 2500 × 608 [14,15]. The metagenes represent a cluster of genes with similar expression values across samples and thus can be considered a cluster of co-expressed or co-regulated genes. Each metagene profile (row-wise) can be interpreted as the mean profile averaged over all gene profiles referring to the respective metagene cluster. Consequently, the metagene values (column-wise) for a single sample represent the metagene expression state for a given sample (sample SOMs heatmap portrait). The profiles of adjacent metagenes are positively correlated while distant metagenes are often negatively correlated.

SOM heatmaps were visualized by arranging them into a two-dimensional 50 × 50 grid, with colors ranging from maroon to blue representing maximum to minimum expression values in each portrait. Due to the SOM’s self-organizing properties, perturbed metagenes form larger clusters, referred to as “spots of modules”. These sample-specific spots are transferred to a global expression or variance summary map, enabling direct comparisons between samples or groups.

Functional annotation of spot genes was performed using Fisher’s Exact test against a gene set collection available in the “oposSOM” package [12], as well as using gProfiler online tool [16].

Finally, sample portraits were stratified into pattern types (PATs), where a PAT is defined by the combination of spot modules co-occurring in the sample portraits as described elsewhere [17]. PAT-specific mean portraits were generated by averaging the portraits of all cases belonging to a particular PAT.

### 2.3. Phenotype Maps and Survival Analysis

We generated SOMs phenotype portraits [18,19] to associate changes in the CLLmap SOMs expression landscape with clinical characteristics obtained from the original publication [7]. The following clinical parameters were used for phenotyping: (1) gender (male/female), (2) CLL molecular subtypes (mutated IGHV, M-CLL/unmutated IGHV, U-CLL), (3) vital status (dead/alive), (4) prior and (5) current treatment types (treated/untreated), (6) immunoglobulin light chain (IGL) expression (Kappa/Lambda/Kappa and Lambda), (7) IGLV3-21^R110^ mutation expression (Yes/No), (8) CLL epigenetic subtype (naive-like, n-CLL/intermediate, i-CLL/memory-like, m-CLL), (9) overall survival (OS), and (10) failure-free survival (FFS).

Phenotype maps were generated by creating a linear regression model with metagene profiles as a dependent variable and clinical characteristic as an independent categorical variable. To generate phenotype portraits for all categories, we used a model without intercept. Then, the corresponding regression coefficients were mapped to the metagene coordinate on the SOMs grid for each metagene. The coefficients were visualized using the same color gradient as the gene SOMs [18,19]. The regression between spot expression and clinical characteristics was performed similarly to the metagene regression. We considered the association significant if the *p*-values of the regression coefficients were less than 0.05. Overall (OS) and failure-free (FFS) survival analyses were performed using gender, spot I activation (binary active/inactive), and PAT type using survival, survminer, and ggplot2 R packages. Cox regression was used to calculate the hazard ratio (HR), and Kaplan–Meier estimate plots were used to visualize survival curves.

### 2.4. Patients and Sample Collection

In this study, we recruited 8 patients with CLL (mean age: 67 ± 5 years, 3 males/5 females) admitted at the Hematology Center named after Professor R.O. Yeolyan. The detailed clinical and demographic parameters are provided in Supplementary Table S1. Morning fasting peripheral venous blood samples were collected in K3EDTA tubes (5 mL) and immediately transferred to the laboratory for RNA isolation.

The study was approved by the Ethics Committee of the Institute of Molecular Biology of the National Academy of Sciences of the Republic of Armenia (IRB#: 00004079, Protocol #: 4/2022, 14 June 2022). All subjects involved in this study provided informed consent.

### 2.5. RNA Isolation

Total RNA was isolated from buffy coat using a Quick-DNA/RNA Miniprep Plus Kit (Zymo Research, Irvine, CA, USA), according to the manufacturer’s instructions. RNA concentration was measured on a Qubit fluorimeter using a Qubit RNA HS kit (ThermoFisher Scientific, Waltham, MA, USA). Samples were stored at −86 °C until further use.

### 2.6. Sequencing Library Preparation

RNA sequencing library for nanopore sequencing was generated using a PCR-cDNA barcoding kit (SQK-PCB109, Oxford Nanopore Sequencing, Oxford, UK) according to manufacturer instructions. Temperature profiles and timings were set according to the protocol. A total of 100 ng of RNA was used as the input for the library preparation. RNA was reverse transcribed with VN and Strand-Switching Primes, Maxima H Minus Reverse Transcriptase (ThermoFisher Scientific, Waltham, MA, USA), and an RNase inhibitor (Invitrogen, Carlsbad, CA, USA). Full-length transcript selection and barcoding were performed with PCR. The reaction mix contained 5 uL of reverse-transcribed RNA, Barcode Primers, and LongAmp Taq Master Mix (New England Biolabs, Ipswich, MA, USA).

The amplified cDNAs were purified using AMPure XP Beads (Beckman Coulter, Brea, CA, USA), with each wash performed using freshly prepared 70% ethanol (at each washing stage). The purified cDNAs were then eluted in 12 uL of Elution Buffer. cDNA concentration in samples was measured with the Qubit dsDNA HS kit (ThermoFisher Scientific, Needham, MA, USA).

For sequencing, the final barcoded cDNA samples were pooled after quantification and a Rapid Adapter was added to our final library, followed by incubation. The final cDNA library of 63 ng was loaded in an R9.4 flow cell for sequencing on the Oxford Nanopore MinION. The run duration was 24 h. Sequencing quality metrics are provided in Supplementary Table S1.

### 2.7. ONT Sequencing Data Preprocessing

Raw data files in FAST5 format were generated with the MinKnow software version 24.11.8. Basecalling and quality filtering were performed with Guppy version 6.01. The resulting FASTQ files were aligned to the reference genome (GRCh38) using minimap2 version 2.24-r1122 with the splice alignment option. After alignment, raw read counts were calculated from the aligned BAM files using the featureCounts function from the Rsubread package [20]. The Gencode v44 GTF file was used for gene annotation. Raw read counts were then transformed into TPM values [21].

### 2.8. Projection of ONT Gene Expression Data to SOMs Space

First, low-expressed genes (TMP < 2 in 50% of samples) were removed from the ONT-CLL and CLLmap datasets. After filtering, 3641 genes were retained in the ONT-CLL dataset and 11,322 in the CLLmap dataset. The CLLmap dataset was further modified to keep only genes that overlap with the ONT dataset, resulting in a total of 3046 genes used for the final analysis. After two datasets were merged, we performed batch effect adjustment using ComBat method [13]. Next, the batch-adjusted dataset was quantile normalized to ensure identical frequency distributions of expression values in all samples (Supplementary Figure S2). Then, we used supSOM as a transfer learning method [22] to project nanopore sequencing samples to SOMs space created with the CLLmap dataset. supSOM adds a support vector machine regression model (SVRM) on top of the original SOMs algorithm to “predict” the portrait of each of the ONT samples. SVRM training used the CLLmap gene expression profiles as independent variables and the corresponding SOMs metagene profile served as a dependent variable. The radius for inclusion of genes [22] in the model was defined as equal to 4 adjacent metagenes. We split the CLLmap dataset into 90% train and 10% validation set. Model performance was assessed using the caret R package. Next, the gene profiles of the corresponding metagene values of the ONT samples were predicted. The PAT assignment of ONT samples was conducted based on the correlation of red and blue channels on SOMs images between ONT samples and PAT group SOMs portraits.

## 3. Results and Discussion

### 3.1. Transcriptome Portrayal of CLL

We used self-organizing maps (SOMs) transcriptome portrayal to perform a molecular stratification of CLL using publicly available RNA-seq data [7]. Our primary goal was to assess CLL molecular diversity and its association with disease clinical features. We used the oposSOM R package to perform dimensionality reduction, clustering, visualization, downstream feature extraction, and diversity analysis [12]. Briefly, the SOMs algorithm transformed a high-dimension gene expression matrix into a reduced-dimensionality matrix of metagenes representing clusters of genes with similar gene expression profiles across the samples in terms of Euclidean distance metric [14,15]. In this way, the intrinsic data structure is unchanged and unaffected by the class labels of the samples (unsupervised learning). Metagenes with close profiles arranged in proximity on the SOMs grid form so-called gene spots or modules that represent a collection of co-expresses or co-regulated genes [14,15]. These spots served as a basis for downstream bioinformatics analysis and functional annotation.

**Figure 1:**
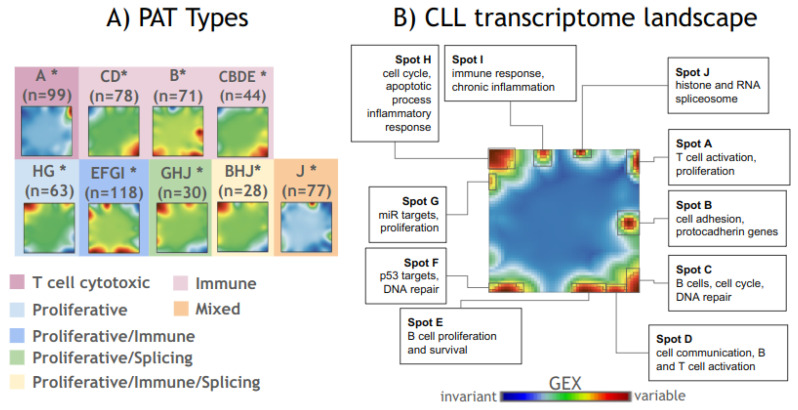
CLL transcriptome landscape. The transcriptome landscapes of CLL samples were generated using the self-organizing maps (SOMs) algorithm, which performs dimensionality reduction and clusters genes into co-expressed gene modules (spots) on a two-dimensional grid. These modules are visualized using a color gradient: blue for underexpression, green for invariant expression, and red for overexpression. Samples with similar gene expression profiles exhibit the same pattern of gene module expression. This key feature of the SOMs algorithm enables the stratification of samples into pattern (PAT) types, based on the similarity of over- and underexpressed gene modules in their SOMs portraits (**A**). Since gene modules (spots) represent clusters of co-expressed genes, their biological functions can be inferred through functional annotation using enrichment analysis, allowing the assignment of biological functions to specific gene modules (**B**).

We used SOMs transcriptome portrayal to perform a class discovery in the CLLmap dataset. CLL samples were stratified according to the pattern (PAT) types defined as a combination of over- and underexpressed spots in each sample [18]. A total of nine PAT types were discovered, each containing 28–118 samples. The averaged group portraits for PAT classes showed considerable variance in activation of gene spots across groups (Figure 1A). The gene spots across PAT classes were then aggregated into a summary map (Figure 1B). This map contained a total of 10 gene spots that were labeled with letters A–J (for a full list of spot-associated genes see Supplementary Tables S2–S11). Each spot represents a module of co-expressed genes with a specific expression profile across samples. On average, they contained 370 genes (min–max: 58–1111 genes). The functional context of the spots was assessed with over-representation analysis (ORA) using a hypergeometric test (Supplementary Table S12).

Overall, the spots were assigned to the two major biological process types: immune response and proliferation. Spots A, B, C, D, E, and I were associated with immune response and were enriched with gene sets related to T and B cell proliferation, activation, and survival, as well as MHC-I-associated genes. These spots also contained signaling pathway signatures, such as MAPK, Rab/Ras, and Wnt signaling. Notably, spot I included gene sets associated with immune responses, chronic inflammation, and signal transduction. Moreover, it contained genes linked to CLL prognosis, such as ZAP70 [23], INSR [24], CLLU1 [25], CRY1 [26], and others, as well as gene sets associated with treatment resistance in other cancers [27,28,29] (Supplementary Figure S3).

Proliferation-related gene modules (spots F, G, and H) of the CLL transcriptome landscape represented with functions such as cell adhesion, p53 targets, DNA repair, RNA synthesis and processing, the MHCII complex, protein deubiquitination, chromatin-modifying enzymes, and proliferative signal target genes (*ESR1*, *CTNNB1*, *TGFB1*).

Spot J showed a unique pattern of gene set enrichment associated with spliceosome (non-coding RNAs) and histone genes.

Next, we performed a co-occurrence analysis [17] to associate PAT types and spots and assign molecular phenotypes (Supplementary Figure S4). According to the molecular context of the spot modules and their combinations, we stratified the PAT subtypes into several subtypes: T-cell cytotoxic (A*), immune (CD*and B*, CBDE*), proliferative (HG*), splicing (J*), and three mixed types: proliferative–immune (EFGI*), proliferative–splicing (GHJ*), and proliferative–immune–splicing (BHJ*).

Thus, the characterization of the transcriptome landscape in CLL with the SOMs portrayal method allowed the identification of different molecular subgroups of CLL characterized by various degrees of the involvement of immune response, proliferation, and splicing.

### 3.2. Phenotype Maps Associate Transcriptome Deregulations with Clinical Characteristics

**Figure 2:**
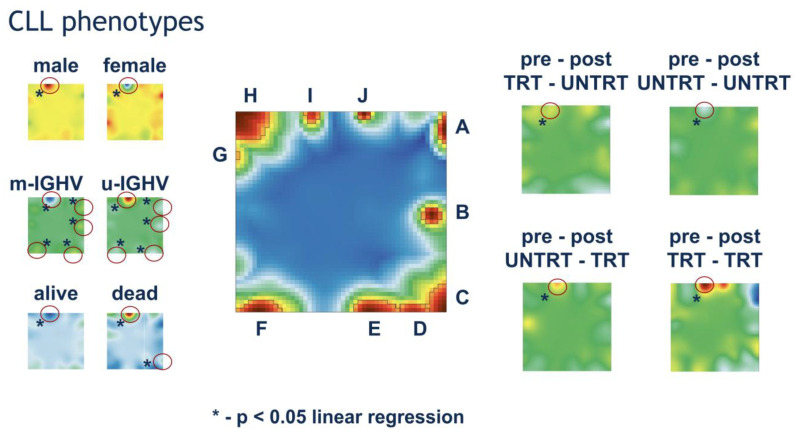
Association of CLL phenotypes with the transcriptomic landscape. Phenotype maps were generated using a linear regression model, where metagene profiles served as the dependent variable and clinical characteristics were used as independent categorical variables. The regression coefficients were visualized to represent the strength and direction of these associations. Left Panels: (1) Gender, (2) CLL molecular subtypes, and (3) Vital status. Right Panels: Prior and current treatment status (treated/untreated). Central Panel: The SOMs transcriptomic landscape of CLL samples, where different spots represent clusters of co-expressed genes. By comparing the locations of these spots with the phenotype maps (red circles), the association between transcriptomic regions and clinical characteristics can be visually assessed. Coloring of phenotype maps is based on regression coefficients, indicating the strength and direction of associations between transcriptomic spots and clinical parameters. Asterisks (*) denote statistically significant associations (*p* < 0.05, linear regression). The results highlight spot I as the most strongly associated with prognosis, gender, IGHV mutation status, and treatment history. Other spots are primarily linked to IGHV mutation status, though to a lesser extent.

We further evaluated the association of PAT types and spots with clinical characteristics in the CLL cohort (Figure 2) using the SOMs phenotype portrayal technique described in detail previously [18,19].

Most clinical parameters showed a strong association with the expression of spot I, which collects immune response and poor prognosis-associated gene sets. Increased expression of this spot was associated with male CLL patients, unfavorable prognosis (death), the non-mutated molecular subtype of CLL, and a naive-like epigenetic phenotype.

In addition to spot I, CLL molecular subtypes were associated with immune response (spots A, B, D) and p53/DNA repair gene sets (spot F). The increased expression of these spots was observed in IGHV-mutated CLL, whereas they were underexpressed in unmutated subtypes. IGL kappa chain expression was positively associated with spot I, while lambda chain expression showed a negative association.

The n-CLL epitype was negatively associated with spots A, B, and D (T cell response/immune response) and positively associated with spot I, whereas this pattern was reversed in the m-CLL epitype. The i-CLL epitype had intermediate expression values, closer to those of m-CLL (Figure 2).

The observed effect of spot I on treatment was remarkable. In the CLL dataset, 15 samples received prior therapy and no treatment after sampling, 215 samples received no prior therapy but were treated after sampling, 353 samples received no prior or post-sampling therapy, and 25 samples received both pre- and post-sampling treatment. The expression of spot I was highest in samples with continuous treatment, followed by samples that started receiving treatment after sampling. On the contrary, samples that received pre-sampling but not post-sampling treatment and samples without therapy had the lowest expression of this spot (Figure 2).

So far, the results indicate that PATs, spot I expression, and gender were strongly related to the CLL phenotypes. Thus, we performed an overall (OS) and failure-free (FFS) survival analysis with the mentioned variables. All three factors showed significant association with survival: (i) T cell and Immune PAT types had a better prognosis compared with mixed and proliferative subtypes (OS *p* < 0.0001, FFS *p* = 0.0008); (ii) females showed significantly better overall and failure-free survival than males (OS *p* = 0.0016, FFS *p* = 0.0008); (iii) low expression of the spot I was associated with a significantly better prognosis than high expression (OS *p* < 0.0001, FFS *p* = 0.0001) (Supplementary Figure S5).

**Figure 3:**
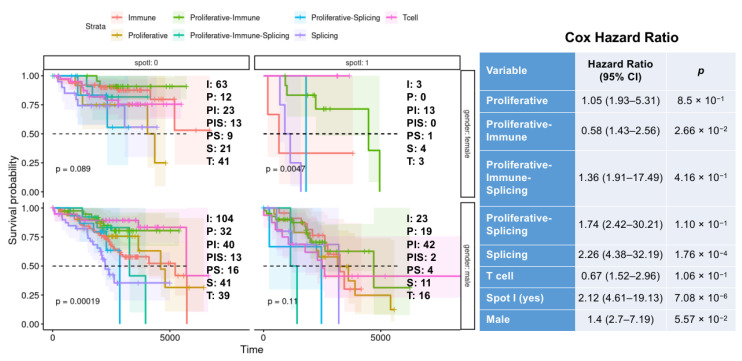
The effect of the interaction between CLL PAT types, spot I expression, and gender on OS. The upper-left panel shows the OS for CLL subtypes in female patients with downregulated spot I; the upper-right panel shows the OS for CLL subtypes in female patients with upregulated spot I; the lower-left panel shows the OS for CLL subtypes in male patients with downregulated spot I; the lower-right panel shows the OS for CLL subtypes in male patients with upregulated spot I. The number of samples per PAT type is indicated near each panel. The PAT type abbreviations are as follows: I—immune; P—Proliferative; PI—Proliferative–Immune; PIS—Proliferative–Immune–Splicing; PS—Proliferative–Splicing; S—Splicing; T—T cell cytotoxic. The association between PAT types, spot I expression, and gender with OS was evaluated using a multivariate Cox regression model. The results are presented as hazard ratios (HRs).

**Figure 4:**
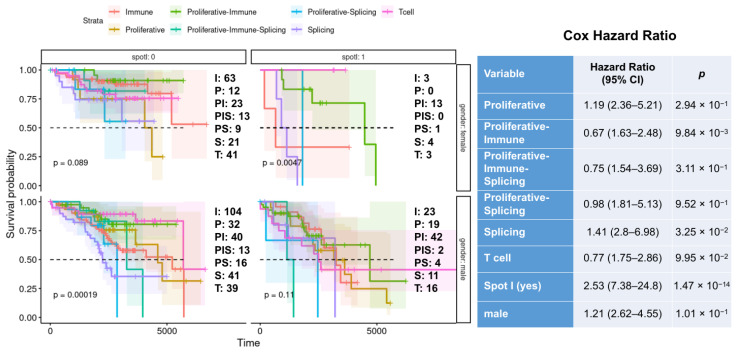
The effect of the interaction between CLL PAT types, spot I expression, and gender on FFS. The upper-left panel shows the FFS for CLL subtypes in female patients with downregulated spot I; the upper-right panel shows the FFS for CLL subtypes in female patients with upregulated spot I; the lower-left panel shows the FFS for CLL subtypes in male patients with downregulated spot I; the lower-right panel shows the FFS for CLL subtypes in male patients with upregulated spot I. The number of samples per PAT type is indicated near each panel. The PAT type abbreviations are as follows: I—immune; P—Proliferative; PI—Proliferative–Immune; PIS—Proliferative–Immune–Splicing; PS—Proliferative–Splicing; S—Splicing; T—T cell cytotoxic. The association between PAT types, spot I expression, and gender with FFS was evaluated using a multivariate Cox regression model. The results are presented as hazard ratios (HRs).

Further stratification based on three factors combined showed notable patterns (Figure 3 and Figure 4). No OS and FFS differences were observed across PAT types in females with low expression of spot I. On the contrary, male patients with low expression of spot I belonging to T cell PAT (pat A*) showed the highest OS and FFS survival, compared to other PAT types. Additionally, females with high expression of the spot I belonging to T cell and proliferative–immune PAT types had a significantly better prognosis than the rest of the PAT types. Finally, no differences in PAT-dependent survival were observed in males with high expression of spot I.

### 3.3. Projection of ONT CLL Data onto CLL SOMs Space

The results of SOMs space annotation allowed the stratification of CLL samples according to their spot profiles (PAT types), functionally annotating them, and associating them with the clinical parameters. Our analyses connected perturbations in the transcriptome levels with the phenotypic characteristics of the CLL. This created feature-label relations that can be used for assigning phenotypes to new samples using machine learning.

In this study, we conducted nanopore sequencing of eight blood samples of first-time CLL patients. The blood was collected immediately upon admission and before treatment. The average initial RNA concentration in the samples was 22.91 ± 18.69 ng/µL. We noted a strong variability in the number of readings per sample (mean library size: 64,444 ± 62,358) (Supplementary Figure S6).

The distribution of gene expression (number of reads per gene) in the samples was typical for RNA sequencing data when most genes were either non-expressed or low-expressed.

**Figure 5:**
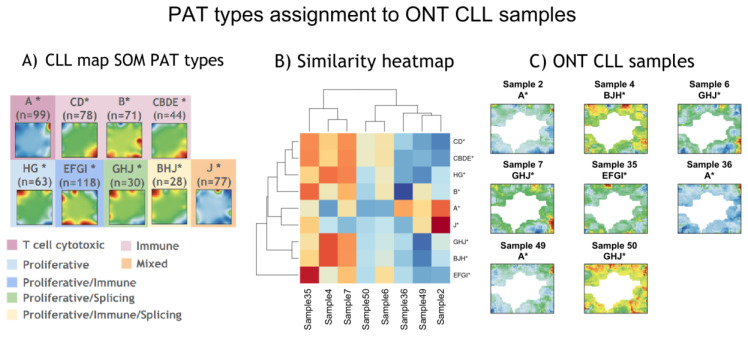
Assignment of PAT types to ONT-CLL Samples. (**A**) Mean PAT group SOMs portraits were generated using the SOMs algorithm based on the public short-read CLLmap RNA-seq dataset (for details, see Figure 1 legend). (**B**) PAT type assignment of ONT samples based on similarity to the CLLmap dataset. The similarity was assessed using Pearson’s correlation, comparing the red and blue channels of SOMs images between projected SOMs portraits of ONT samples and PAT-type SOMs portraits. The heatmap represents correlation coefficients between mean CLLmap PAT subtypes and ONT samples. Each ONT sample was assigned to the PAT type with the highest correlation coefficient. (**C**) SOMs images of ONT samples generated using the supSOM algorithm, which applies a support vector regression model trained on CLLmap gene expression profiles (training set) to classify ONT samples (test set). White areas in ONT sample SOMs images correspond to metagenes that did not contain genes from the common gene set (3641 genes) shared between the ONT and CLLmap datasets.

To perform phenotype assignment for the samples in our ONT cohort, we used a previously developed SOMs projection method known as supSOM, described in detail elsewhere [22]. The supSOM model was trained based on the reduced set of genes present in both CLLmap and ONT RNA-seq datasets (3641 genes). Before proceeding to the projection of ONT-CLL data, we checked the model’s performance based on CLLmap data. The results showed perfect correspondence between mean portraits of the original and predicted PAT types (Supplementary Figure S7). The multi-class model performance on the validation dataset was as follows: an overall sensitivity of 0.58, specificity of 0.94, and balanced accuracy of 0.76. A receiver operating characteristic (ROC) curve was generated for one-versus-all classification of PAT subtypes, with area under the curve (AUC) values ranging from 0.58 to 0.99 (Supplementary Figure S8). Next, we proceeded to the prediction of PAT subtypes for nanopore-sequenced samples. SupSOM projection distributed eight ONT CLL samples into four PAT types (Figure 5). Six samples of eight were placed on the wait-and-watch treatment since no symptoms were observed (Supplementary Table S1). Two male patients assigned to EGFI* (Sample 35, proliferative–immune) and GHJ* (Sample 6, proliferative–splicing) received post-admission treatment. Furthermore, the EGFI* patient (Sample 35) demonstrated overexpression of spot I—a transcriptomic hallmark of unmutated IGHV. Moreover, this patient also had a somatic loss of *TP53* allele identified by molecular–genetic analyses, which is frequent in unmutated CLL [30].

## 4. Discussion

Using a molecular portrayal technique [14], we dissected the CLL transcriptome landscape into deregulated functional gene modules associated with T cell cytotoxicity, B and T cell activation, inflammation, cell cycle, DNA repair, proliferation, and splicing. These findings conform with the previous knowledge on gene expression deregulation in CLL [5]. Based on the perturbation of these modules in CLL samples, we identified several transcriptomic subtypes of CLL characterized by distinctive activity profiles of functional gene modules. Notably, genes previously associated with CLL prognosis were colocalized in a single gene module and correlated with genetic and epigenetic classification, gender, and treatment response. However, this gene module was not strongly associated with CLL transcriptomic subtypes, suggesting that molecular subtypes of CLL extend beyond genetic and epigenetic classifications [5,7,31]. Moreover, the balance between the expression of immune, proliferation, and splicing signatures in these molecular subtypes directly impacted the survival outcomes. T-cell activation was associated with the most favorable prognosis [32,33] while splicing signatures correlated with poorer outcomes [34].

Gender is a known risk factor in CLL, with higher incidence and generally more aggressive progression in males than in females [35]. We observed that the interaction between gender and molecular subtypes significantly influenced survival in CLL. While our results confirmed previous findings, they also revealed that specific molecular subtypes and associated gene expression patterns impact gender-related survival differences. These differences were evident only in the immune mixed subtypes, while no survival differences were observed for the most favorable (T-cell activation) and other subtypes (Supplementary Figures S9 and S10). These findings demonstrate that existing risk factors and molecular subtype classifications do not fully capture disease diversity, highlighting the value of transcriptomic subtyping as an additional, independent layer for improved prognosis and monitoring.

Another outcome of our study is demonstrating the potential for assigning molecular subtypes and predicting clinical outcomes using nanopore sequencing. Nanopore sequencing is being actively explored as a cost-effective diagnostic and prognostic tool, with applications in genetic testing [36], and rapid detection of bacterial [37] and viral pathogens [38]. All these use cases are based on the detection and evaluation of DNA, which, given sufficient nanopore sequencing depth, works and gives adequate sensitivity. However, the use of nanopore sequencing for transcriptomics-based classification remains scarce, though with promising results. For example, a recent study demonstrated the potential of nanopore RNA sequencing with machine learning to classify acute leukemias [11]. Challenges in nanopore-based classifications stem from the inherent advantages of the technology. The primary benefit of nanopore sequencing is its ability to cost-effectively process small numbers of samples at a time, making it suitable for resource- and sample-flow-limited settings. Sequencing cost breakdown showed that 12 samples can be sequenced for cca. USD 140 per sample (Supplementary Table S13) without significant investment in infrastructure and a large number of samples per run requirement. However, this results in relatively small datasets within individual laboratories, complicating classifier training. Additionally, nanopore RNA sequencing generates significantly less data compared to Illumina platforms, and no nanopore-based equivalents to large repositories like The Cancer Genome Atlas [39] currently exist. Furthermore, lower sequencing depth and accuracy of nanopore platforms limit clinical decision making based solely on their data. While nanopore sequencing accuracy is primarily evaluated in DNA sequencing applications [40], it can also impact RNA sequencing results. Errors introduced during nanopore sequencing can lead to misalignments, ultimately affecting gene quantification. Additionally, the lower throughput of ONT sequencing restricts the reliable quantification of only relatively highly expressed transcripts. This is evident in this study, where filtering out low-expressed transcripts retained approximately 3000 transcripts in the ONT-CLL dataset compared to around 11,000 in the CLLmap dataset. Consequently, many relevant genes may not be detected using nanopore sequencing, which can limit the accurate interpretation of ONT-derived transcriptomic data. Therefore, additional strategies are required to address these limitations, including data harmonization approaches, as nanopore data are not directly compatible with short-read Illumina data. Here, we applied a previously developed machine learning approach [22] to enhance transfer learning and integrate clinically relevant information into nanopore-sequenced samples. First, the SOMs algorithm arranges genes into metagenes and modules based on their co-expression/co-occurrence patterns [14,15]. Since the SOMs is trained on Illumina data, it provides expression profiles for a much larger set of genes, assigning ONT-derived genes to a metagene using supSOM allows us to infer the expression patterns of other genes associated with the same module. This approach helps compensate for missing data in nanopore sequencing and enables a more comprehensive interpretation of transcriptomic perturbations.

Through supSOM training, we associated Illumina input data with identified transcriptomic subtypes and successfully assigned those to nanopore-sequenced samples. This approach resolved data compatibility issues and enabled the transfer of rich functional insights from public short-read datasets to nanopore data. We believe that this strategy has significant potential for tailoring treatment strategies and improving patient management in resource-limited settings.

A notable limitation of this study is the small number of nanopore-sequenced samples. A larger sample group will be necessary to evaluate classification performance more accurately. Nonetheless, the results are promising and warrant further exploration.

## 5. Conclusions

Our study demonstrates that the CLL transcriptome landscape can be dissected into functional modules that reveal distinct molecular subtypes based on proliferative and immune activity, with significant implications for prognosis and treatment orthogonal to other known molecular subtypes. Furthermore, the integration of nanopore sequencing, public datasets, and machine learning highlights a cost-effective strategy for molecular subtyping and prognostic prediction, supporting more accessible and personalized CLL care.

## Data Availability

The code for reproducing the results and data associated with this study is deposited in the Zenodo open repository (https://zenodo.org/record/14505141/ (accessed on 4 March 2025). The CLLmap project data are available at https://cllmap.org/ (accessed on 4 March 2025). The Nanopore RNA sequencing data will be deposited in the Gene Expression Omnibus (ID: TBD).

## Supplementary Materials

The following supporting information can be downloaded at: https://www.mdpi.com/article/10.3390/cancers17060964/s1, Supplementary Table S1: Characteristics of patients who underwent nanopore sequencing, Supplementary Table S2: Genes associated with gene module (spot) A, Supplementary Table S3: Genes associated with gene module (spot) B, Supplementary Table S4: Genes associated with gene module (spot) C, Supplementary Table S5: Genes associated with gene module (spot) D, Supplementary Table S6: Genes associated with gene module (spot) E, Supplementary Table S7: Genes associated with gene module (spot) F, Supplementary Table S8: Genes associated with gene module (spot) G, Supplementary Table S9: Genes associated with gene module (spot) H, Supplementary Table S10: Genes associated with gene module (spot) I, Supplementary Table S11: Genes associated with gene module (spot) J, Supplementary Table S12: Specific gene sets associated with spots (gene modules), Supplementary Table S13: Cost breakdown for nanopore RNA sequencing, Supplementary Figure S1: Batch adjustment of the CLLmap dataset for downstream analysis, Supplementary Figure S2: Combining ONT-CLL (blue dots) and CLLmap (red dots) datasets for downstream analyses, Supplementary Figure S3: Mapping CLL-associated gene signatures onto the CLLmap SOMs landscape, Supplementary Figure S4: Co-occurrence matrix of PAT types and gene modules, Supplementary Figure S5: Overall (OS) and failure-free (FFS) survival rates depending on gender, spot I expression, and PAT types as independent factors, Supplementary Figure S6: Library size for CLL samples undergoing nanopore sequencing, Supplementary Figure S7: Prediction of CLLmap PAT types using SVMR-SOM, Supplementary Figure S8: Receiver operating characteristic (ROC) curves for one-versus-all classification of PAT subtypes, Supplementary Figure S9: The influence of CLL PAT types on gender-related OS, Supplementary Figure S10: The influence of CLL PAT types on gender-related FFS.
