# Supplementary material for: Assigning Transcriptomic Subtypes to Chronic Lymphocytic Leukemia Samples Using Nanopore RNA-Sequencing and Self-Organizing Maps
Source: Cancers (Basel). 2025 Mar 13;17(6):964. doi: 10.3390/cancers17060964 (PMC11940626; doi:10.3390/cancers17060964)
Supplement: Supplementary file 1 [file cancers-17-00964-s001.zip › Supplementary Figures.pdf]

Assigning transcriptomic subtypes to CLL samples using nanopore RNA-sequencing and self-organizing maps

Supplementary Figures

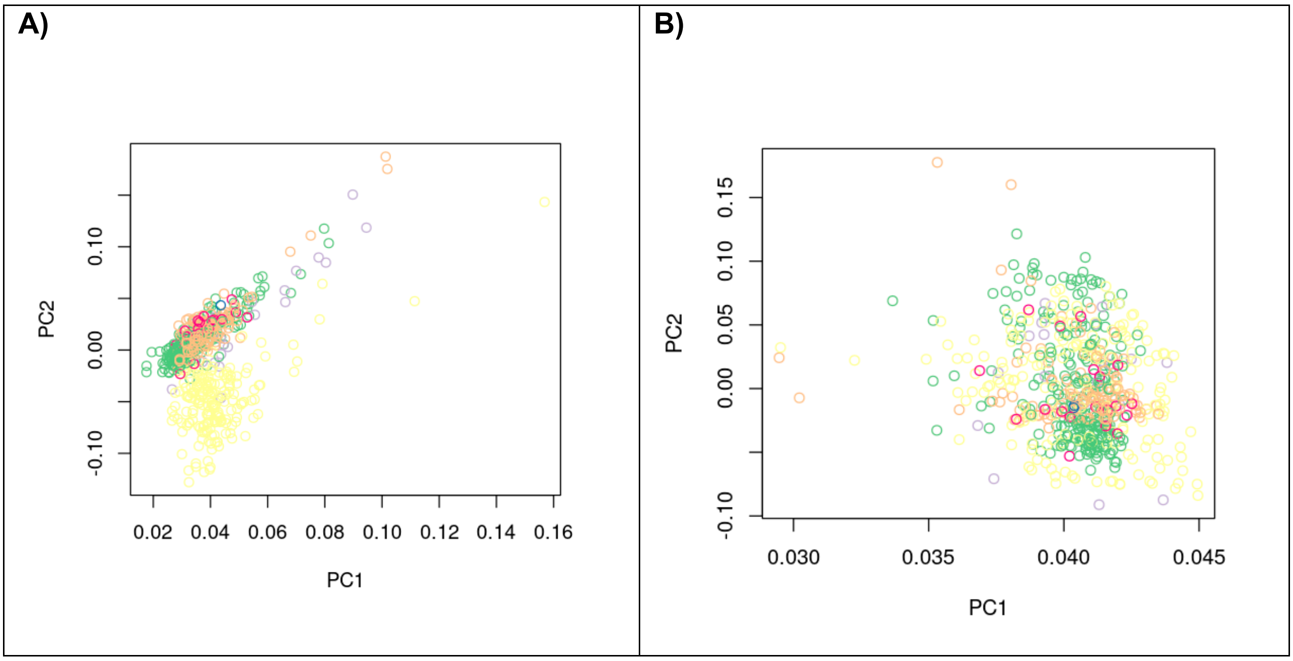

Figure S1. Batch adjustment of the CLL-map dataset for downstream analysis. The CLL-map dataset composed on several cohorts. We performed batch (cohort) adjustment using Combat approach. A) PCA plot before batch adjustment. B) PCA plot after batch adjustment. Colors indicate the dataset batches.

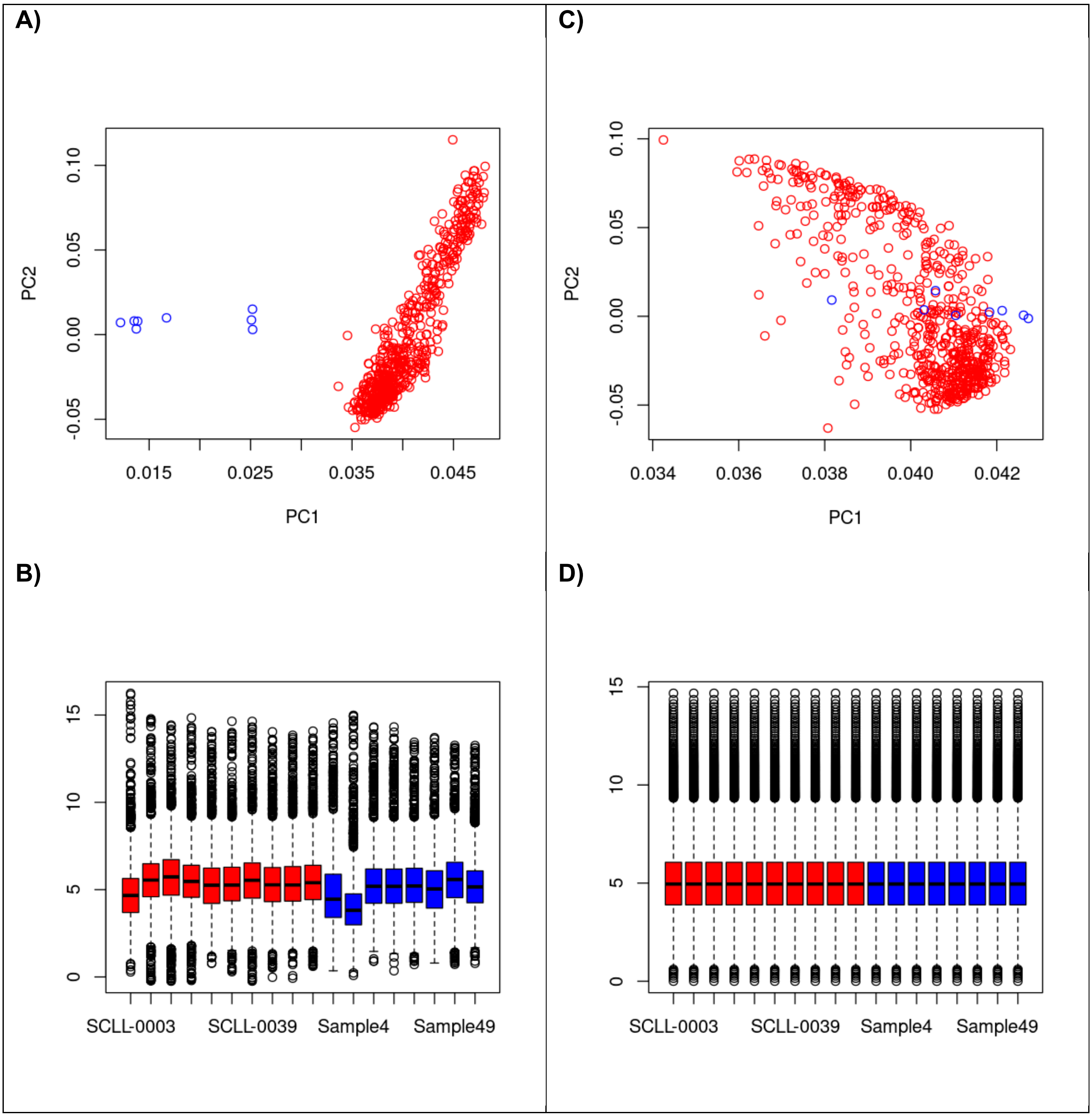

Figure S2. Combining ONT-CLL (blue dots) and CLLmap (red dots) datasets for downstream analyses. A) PCA plot before batch adjustment and quantile normalization. B) Boxplot of gene expression distribution before quantile normalization. C) PCA plot after batch adjustment and quantile normalization, D) Boxplot of gene expression distribution after quantile normalization.

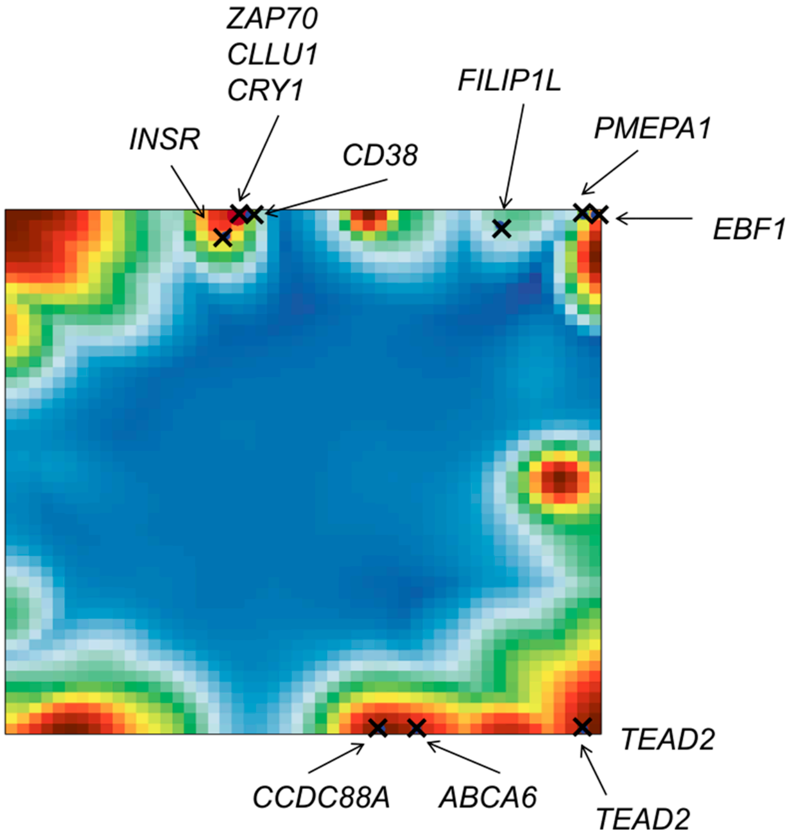

Figure S3. Mapping CLL-Associated Gene Signatures onto the CLLmap SOM Landscape. The gene list was compiled from multiple publications addressing CLL prognosis and treatment-related markers (see references 23–29 in the main manuscript). Genes were mapped onto the SOM landscape based on gene-metagene associations. The presence of these genes within deregulated regions or their close vicinity supports the validity of the identified gene modules, their functional roles, and their association with clinical characteristics.

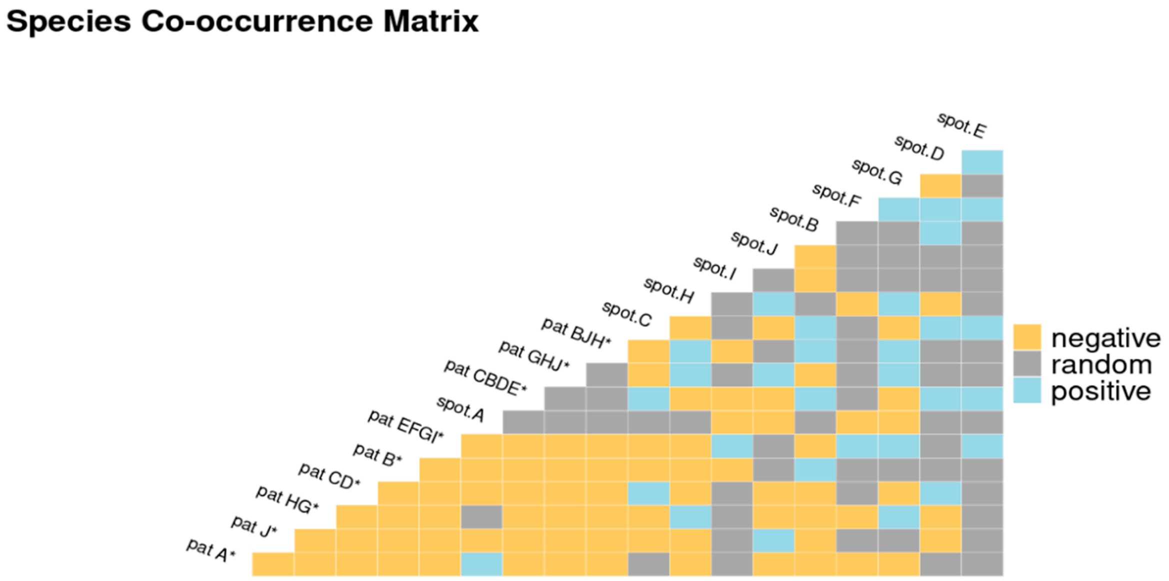

Figure S4. Co-occurrence matrix of PAT types and gene modules. The co-occurrence matrix was calculated based on the observed and expected frequencies of pairwise co-occurrence between PATs and spot modules (For details see Material and Methods section).

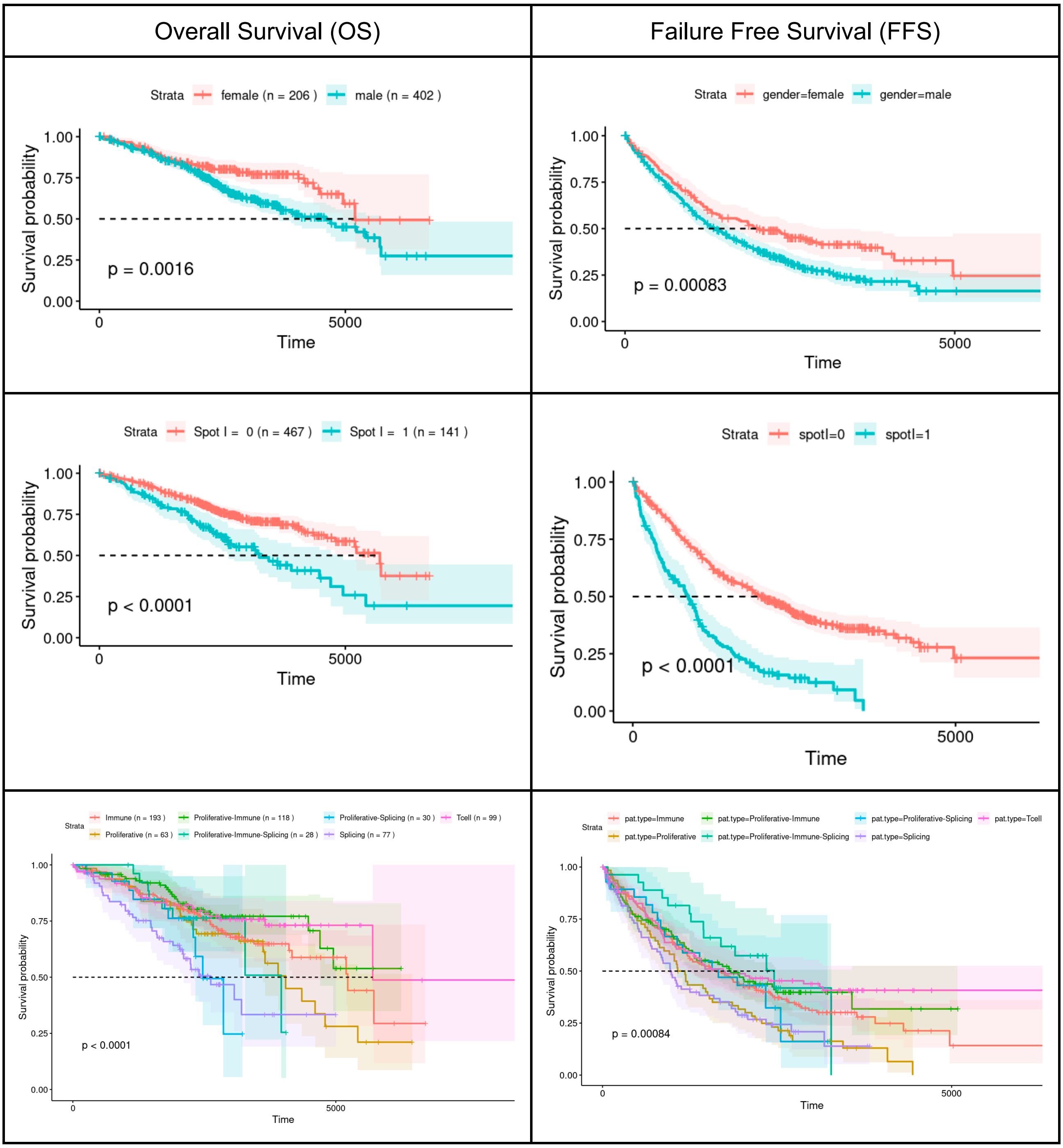

Figure S5. Overall (OS) and Failure-free (FFS) survival rates depending on gender, spot I expression, and PAT types as independent factors.

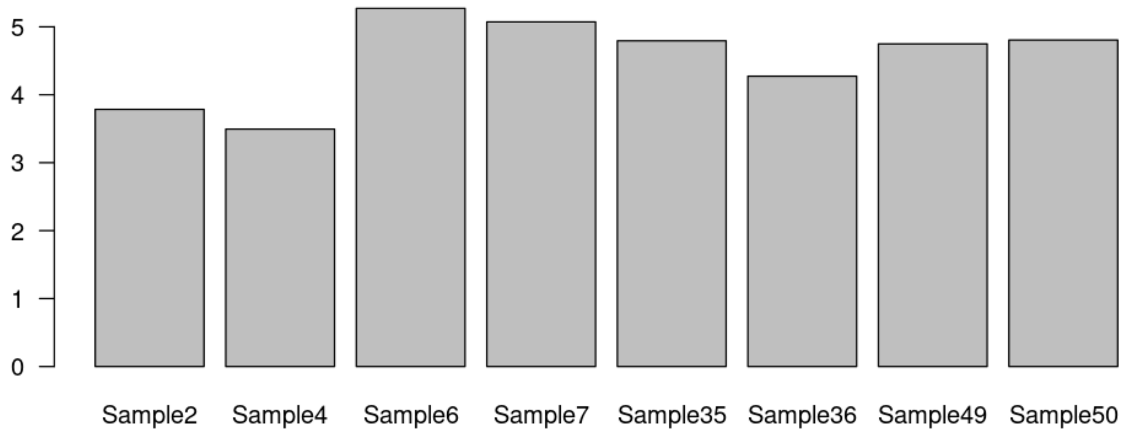

Figure S6. Library size for CLL samples undergone nanopore sequencing.

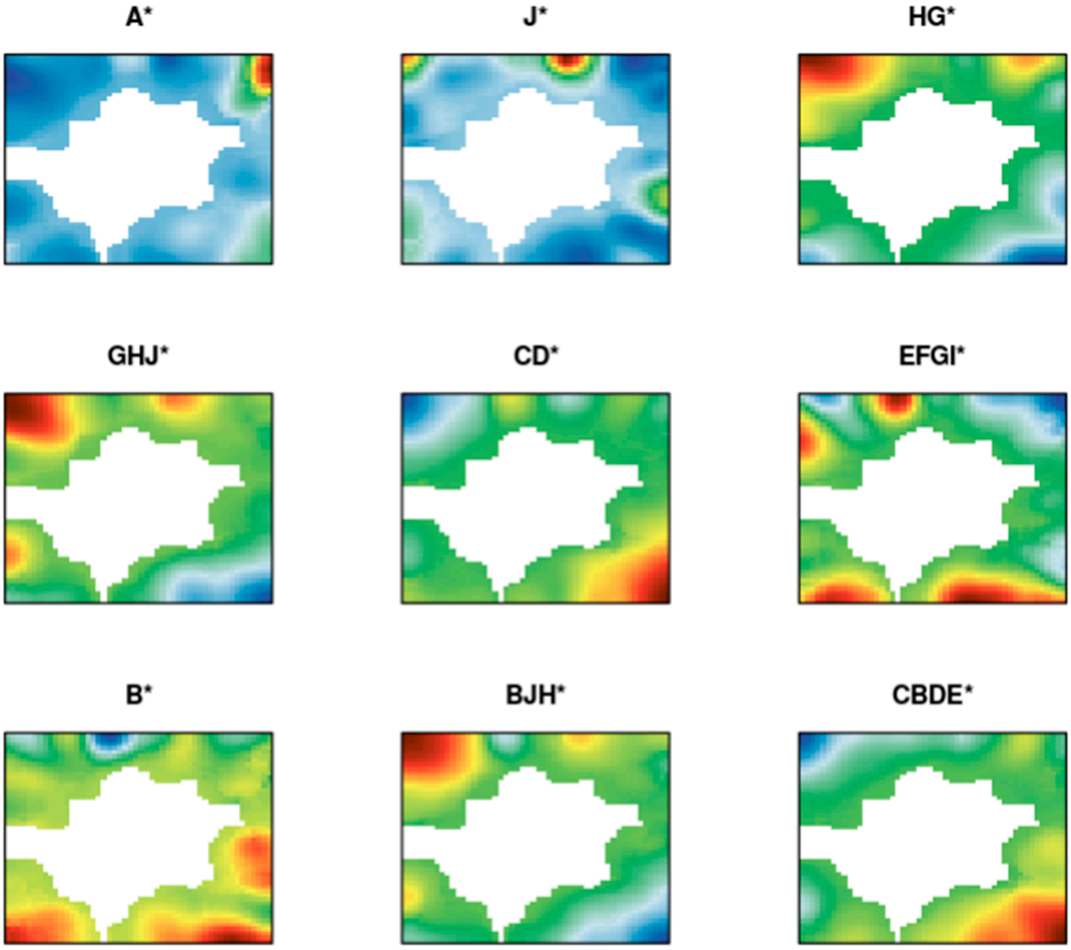

Figure S7. Prediction of CLL map PAT types using supSOM. In total, 3046 gene profiles were used for supSOM analysis. The white areas indicate metagenes where no associated genes were found in the training dataset. These metagenes were assigned "NA" values during training. When comparing the white areas with the original SOM portraits one can notice that these areas on the predicted portrait correspond to the invariant areas on the original SOM portrait of corresponding PAT type.

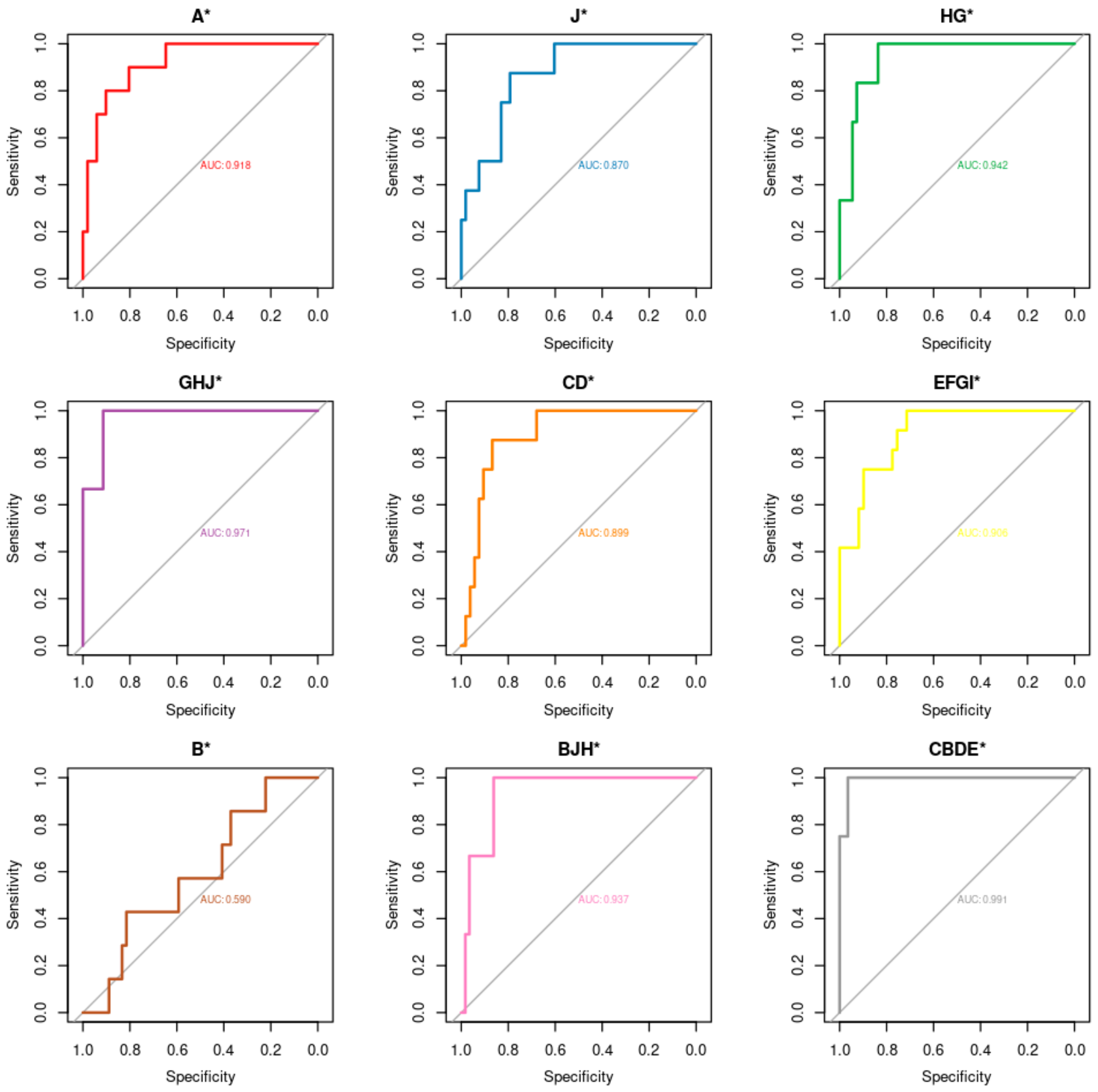

Figure S8. Receiver operating characteristic (ROC) curves for one-versus-all classification of PAT subtypes. pROC R package was used for ROC curves and AUC calculation.

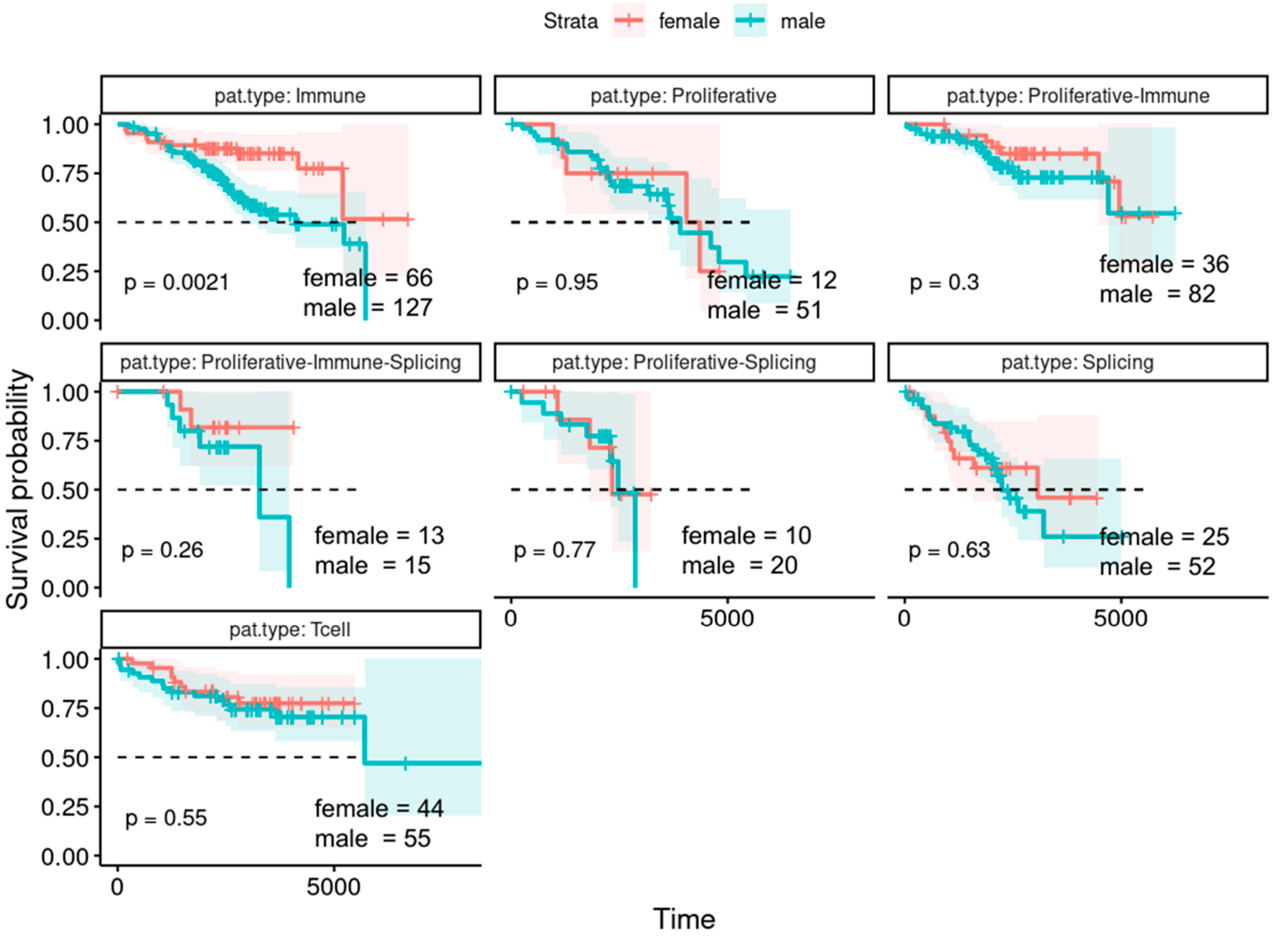

Figure S9. The influence of CLL PAT types on gender-related OS.

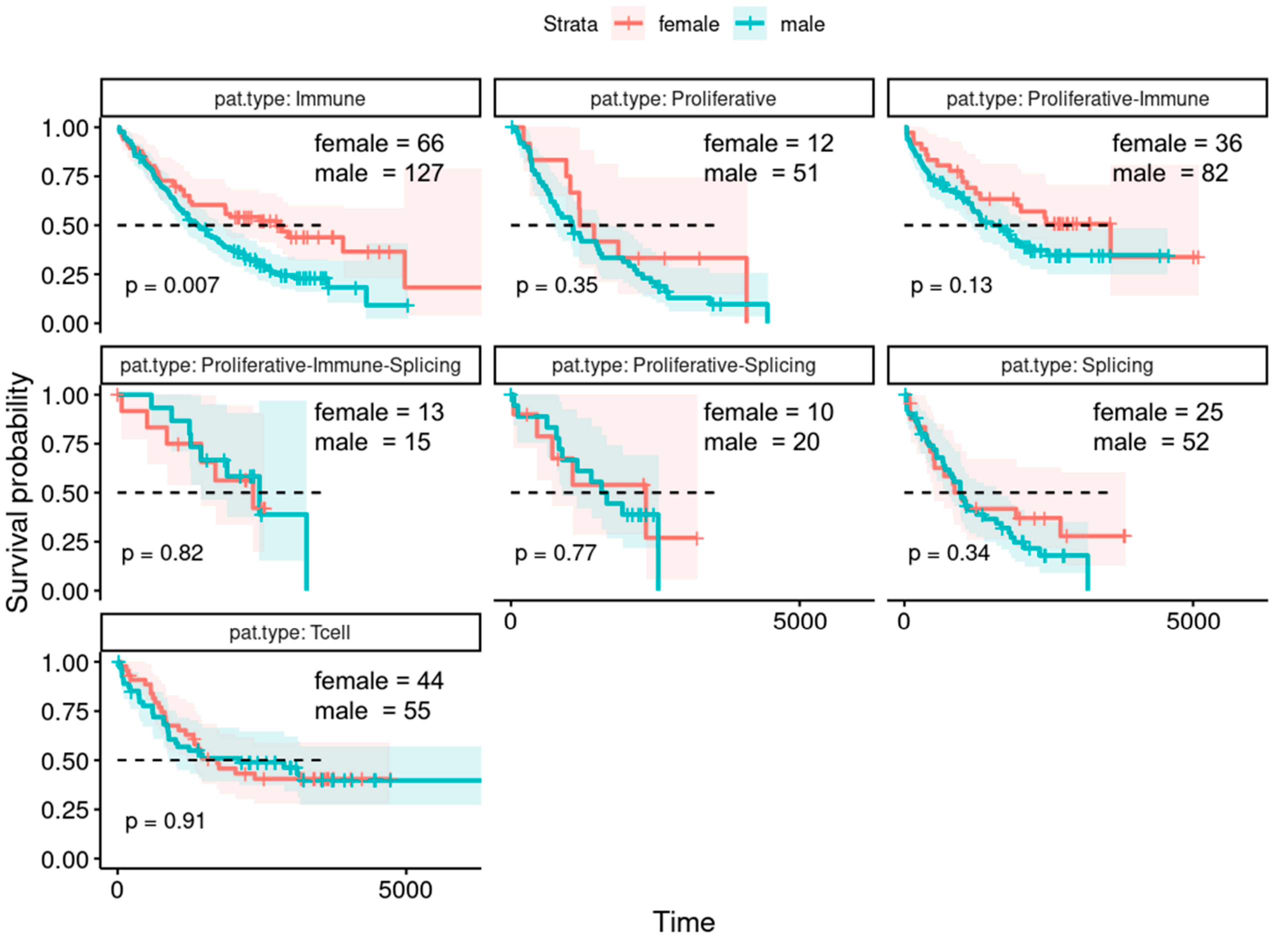

Figure S10. The influence of CLL PAT types on gender-related FFS.
